# 25 Hydroxyvitamin D Deficiency and Its Relationship to Autoimmune Thyroid Disease in the Elderly

**DOI:** 10.3390/ijerph13090850

**Published:** 2016-08-26

**Authors:** Giovanna Muscogiuri, Daniela Mari, Silvia Prolo, Letizia M. Fatti, Maria Celeste Cantone, Paolo Garagnani, Beatrice Arosio, Carolina Di Somma, Giovanni Vitale

**Affiliations:** 1 I.O.S. & COLEMAN Srl, Via Alcide De Gasperi, 107,109,111, Acerra (NA) 80011, Italy; giovanna.muscogiuri@gmail.com; 2 Department of Clinical Sciences and Community Health (DISCCO), University of Milan, Via Della Commenda 9/12, Milan 20122, Italy; daniela.mari@unimi.it (D.M.); prolo@libero.it (S.P.); celeste.cantone@gmail.com (M.C.C.); beatrice.arosio@unimi.it (B.A.); 3 Geriatric Unit, Fondazione IRCCS Ca’ Granda, Ospedale Maggiore Policlinico, Via Pace, 9, Milan 20122, Italy; 4 Division of Endocrine and Metabolic Diseases, Istituto Auxologico Italiano IRCCS, Piazzale Brescia 20, Milan 20149, Italy; l.fatti@auxologico.it; 5 Department of Experimental, Diagnostic and Specialty Medicine (DIMES), University of Bologna, Via San Giacomo 12, Bologna 40126, Italy; paolo.garagnani2@unibo.it; 6 Interdepartmental Center “L. Galvani” (C.I.G.), University of Bologna, Via San Giacomo 12, Bologna 40126, Italy; 7 IRCCS SDN, Napoli Via Gianturco 113, Naples 80143, Italy; cdisomma@unina.it; 8 Laboratory of Endocrine and Metabolic Research, Istituto Auxologico Italiano IRCCS, Via Zucchi 18, Cusano Milanino (MI) 20095, Italy

**Keywords:** vitamin D, autoimmune thyroid disease, elderly, lifestyle, environment

## Abstract

*Background*: Low 25(OH) vitamin D levels have been associated with several autoimmune diseases and recently with autoimmune thyroiditis (AT). The aim of the study was to investigate the association of AT with low 25(OH) vitamin D levels in the elderly. *Methods*: One hundred sixty-eight elderly subjects (mean age: 81.6 ± 9.4 years) were enrolled. Serum levels of 25(OH) vitamin D, anti-thyroid peroxidase (TPO-Ab), anti-thyroglobulin (TG-Ab) antibodies, free triiodothyronine (FT3), free thyroxine (FT4) and thyroid stimulating hormone (TSH) were measured. Results: The prevalence of AT was significantly higher in subjects with vitamin D deficiency (25(OH) vitamin D < 20 ng/mL) when compared with subjects with normal 25(OH) vitamin D (25(OH) vitamin D ≥ 20 ng/mL) levels (28% vs. 8%, respectively, *p* = 0.002). Patients with AT and vitamin D deficiency had a comparable hormonal profile compared to patients with AT and vitamin D sufficiency in terms of TSH (*p* = 0.39), FT3 (*p* = 0.30), FT4 (*p* = 0.31), TG-Ab (0.44) and TPO-Ab (0.35). Interestingly, a significant correlation between 25(OH) vitamin D and TPO-Ab (*r* = −0.27, *p* = 0.03) and FT3 (*r* = 0.35, *p* = 0.006) has been found in subjects with AT while no correlation was found between 25(OH) vitamin D levels and TG-Ab (*r* = −0.15, *p* = 0.25), TSH (*r* = −0.014, *p* = 0.09) and FT4 (*r* = 0.13, *p* = 0.32). *Conclusions*: These findings suggest that vitamin D deficiency was significantly associated with AT in the elderly. Therefore, the screening for AT should be suggested in subjects with vitamin D deficiency.

## 1. Introduction

Humans derive vitamin D from cutaneous synthesis (in the form of cholecalciferol (D_3_)), from diet (in the form of D_3_) and from nutritional supplements in the form of D_3_ or ergocalciferol (D_2_) [1]. Thanks to the exposure to ultraviolet B radiation (UVB), 7-dehydrocholesterol is converted to pre-vitamin D_3_ in the skin, which is in turn converted to vitamin D_3_. After ingestion or synthesis, vitamin D is hydroxylated in the liver by Cytochrome P450 Family 2 Subfamily R Member 1 (CYP2R1) to form 25 hydroxyvitamin D (25(OH)D_2_ or 25(OH)D_3_), its major circulating form, which has little biological activity. The 25(OH)D is converted in the kidney by Cytochrome P450 Family 27 Subfamily B Member 1 (CYP27B1), to its bioactive hormonal metabolite 1,25 dihydroxy-vitamin D (1,25(OH)_2_D or calcitriol). The 25(OH)D 1α hydroxylase has been also found in extrarenal sites including placenta, monocytes and macrophages [1,2]. The 1,25(OH)_2_D binds the nuclear vitamin D receptor, which heterodimerizes with the retinoid X receptor and binds to vitamin D–responsive elements near target genes [3]. The main role of vitamin D is to preserve calcium and phosphorus homeostasis in order to maintain bone health [4]. Recent evidence reported numerous extra-skeletal effects of 25(OH) vitamin D which may also contribute to the pathogenesis of several non-skeletal disorders [5]. In particular, 25(OH) vitamin D has been demonstrated to have a role in thyroid disease. In fact, the vitamin D_3_ receptor and the receptor for the thyroid hormone have been reported to have a similar molecular structure, which was due to two regions that they have in common: the first is a 70-amino-acid, cysteine-rich sequence and the second region is a 62-amino-acid one located towards the carboxyl terminus of the protein [6]. Emerging, although still provisional, evidence also suggests a possible role for 25(OH) vitamin D in the pathogenesis of autoimmune thyroiditis (AT). Vitamin D deficiency, certain vitamin D receptor (VDR) gene polymorphisms, and alterations of vitamin D binding proteins (VDB) and of their genes may be involved in the onset of AT [7]. Gene polymorphisms of numerous proteins and enzymes associated with vitamin D functions, such as VDR, DPB, CYP27B1 and CYP2R1, have been found to be associated with thyroid autoimmunity susceptibility [7,8]. Lower vitamin D levels (<10 ng/mL) have been found to be more common in patients with AT compared to healthy controls [9]. The same conclusions were reached by Tamer et al. [7] reporting, in patients with vitamin D insufficiency (<30 ng/mL), a greater chance to have AT than the healthy population. However, several studies reported negative results regarding the association of vitamin D and AT. In particular, no association between vitamin D deficiency and AT was found by Goswami et al. [10] in a study performed in a population of Asian Indians. Effraimidis et al. [11] reported similar results in a study performed in the Amsterdam AT cohort. This cohort was followed up for five years. At the end of the follow-up no association was found between AT and low vitamin D levels.

In polycystic ovary syndrome, we have demonstrated that the levels of 25(OH) vitamin D were significantly lower in women with Polycystic Ovary Syndrome (PCOS) and AT when compared to women with PCOS and without AT, although no correlation was found between 25(OH) vitamin D and anti-thyroid peroxidase antibodies (TPO-Ab), anti-thyroglobulin (TG-Ab) antibodies, free triiodothyronine (FT3), free thyroxine (FT4) and thyroid stimulating hormone (TSH) levels [12].

The main limit of these studies is that the association of 25(OH) vitamin D with AT has been assessed in young and/or middle-aged subjects. To date, no studies have been performed in the elderly. 

Based on these considerations, the aim of the current study was to investigate the association of AT with low vitamin D levels in the elderly population, where both conditions are quite common.

## 2. Materials and Methods

### 2.1. Subjects

A total of 168 patients aged 65 years or older were retrospectively enrolled in the Division of General Internal Medicine and Geriatrics—Italian Auxologic Institute—from April 2010 to June 2010, after obtaining written consent. In order to avoid seasonal influences on 25(OH) vitamin D levels, the study has been carried out in a short time-period. Exclusion criteria included: age < 65 years; treatment with medications affecting thyroid function (l-thyroxine, antithyroid drugs, antiepileptic, antipsychotics, amiodarone, glucocorticoid, etc.); treatment with vitamin D or calcium supplement; administration of immunomodulatory medications.

### 2.2. Clinical and Biochemical Assessment

For all subjects, body mass index (BMI) was calculated as weight (kg)/(height (m)^2^). After fasting overnight for 10–12 h, blood samples were collected for the following measurements: 25(OH) vitamin D, FT3, FT4, TSH, TG-Ab, TPO-Ab, hepatic and renal chemistries. Subjects with elevated TG-Ab and/or TPO-Ab serum levels performed thyroid ultrasound in order to confirm the diagnosis of AT.

Serum levels of 25(OH) vitamin D were determined through electrochemiluminescence binding assay using the Cobas analyzer (Roche Diagnostics) [13]. Serum TSH, FT3 and FT4 levels were measured by means of electrochemiluminescence immunoassay (Roche Diagnostics). TG-Ab and TPO-Ab measurements were undertaken by electrochemiluminescence immunoassay methods using the Cobas analyzer (Roche Diagnostics). Modular P800 system (Roche Diagnostics) was used for evaluation of creatinine, aspartate aminotransferase, alanine aminotransferase, glucose, total cholesterol and triglycerides.

### 2.3. Definition of AT

AT was defined on the basis of serum TG-Ab (>115 IU/mL) and/or TPO-Ab (>34 IU/mL) positivity, together with characteristic ultrasonographic features (diffuse parenchymal hypoechogenicity and/or heterogeneous echogenic pattern of thyroid gland).

### 2.4. Definition of Vitamin D Deficiency

Vitamin D deficiency was defined as 25(OH)D levels < 20 ng/mL while 25(OH)D levels ≥ 20 ng/mL were considered normal [14]. 

### 2.5. Statistical Analysis 

Statistical analysis was carried out using SPSS 9.0 (Chicago, IL, USA). Data are expressed as mean ± SD (Standard Deviation). Pearson correlations were used to determine relationships between variables. Depending on the distribution of the data, the Student’s *t*-test for independent samples and the non-parametric ManneWhitney U-test for independent samples were applied to test for differences between groups. Pearson correlations were used to determine relationships between variables.

## 3. Results

Clinical and biochemical variables of the entire cohort of patients stratified by 25(OH) vitamin D levels are shown in Table 1. A total of 168 subjects (mean age 81.6 ± 9.4 years) were enrolled in this study. 

Selecting 20 ng/mL as the cut-off point, low 25(OH) vitamin D levels were detected in 70% of all patients. The overall prevalence of AT was 23%. 

We compared the prevalence of AT between patients with vitamin D deficiency (25(OH) vitamin D < 20 ng/mL) and sufficiency (25(OH) vitamin D ≥ 20 ng/mL), finding that AT was significantly more frequent in patients with vitamin D deficiency (28% vs. 8%, *p* = 0.002).

Although the prevalence of AT was higher in patients with vitamin D deficiency, the subjects with vitamin D deficiency did not differ from subjects with vitamin D sufficiency in terms of TSH (2.1 ± 1.9 vs. 1.8 ± 1.3 mcUI/mL, *p* = 0.14), FT3 (3.7 ± 1.0 vs. 4.1 ± 1.6 pg/mL, *p* = 0.16) and FT4 levels (15.3 ± 5.3 vs. 14.7 ± 3.1 ng/dL, *p* = 0.33). As expected, subjects with vitamin D deficiency were significantly older compared to subjects with normal vitamin D levels (82.8 ± 8.4 vs. 78.8 ± 10.8 years, *p* = 0.005). Further, we investigated if patients with AT and vitamin D deficiency (*n* = 21) had a worse hormonal profile compared to patients with AT and vitamin D sufficiency (*n* = 18), but we did not find significant differences between the two groups (TSH: 1.6 ± 0.8 vs. 1.7 ± 1.1 mcUI/mL, *p* = 0.39; FT3: 4.0 ± 0.8 vs. 3.7 ± 1.0 pg/mL, *p* = 0.30; FT4: 15.2 ± 3.0 vs. 14.3 ± 3.3 ng/dL, *p* = 0.31; TG-Ab: 293.7 ± 203.0 vs. 303.6 ± 210.1, *p* = 0.44; TPO-Ab: 252.4 ± 180.0 vs. 229.7 ± 193.3, *p* = 0.35).

**Figure 1:**
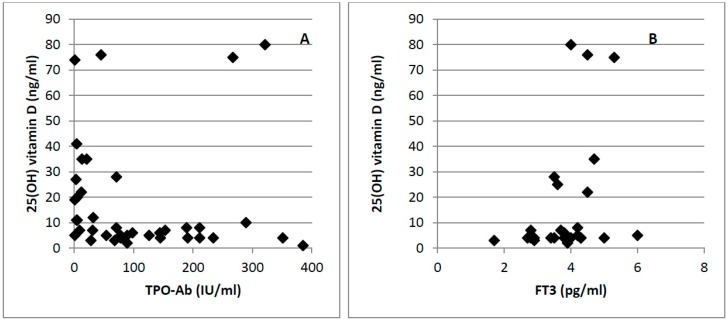
Correlations between 25(OH)D concentration and TPO-Ab (*r* = −0.27, *p* = 0.03) (**A**) and FT3 (*r* = 0.35, *p* = 0.006) (**B**).

In addition, we focused on patients with AT in order to investigate if vitamin D levels were associated with the grade of severity of AT in the overall population. In this subgroup Pearson coefficient analyses revealed a significant correlation of 25(OH) vitamin D levels with TPO-Ab (*r* = −0.27, *p* = 0.03) and FT3 (*r* = 0.35, *p* = 0.006) (Figure 1), while no correlation was found with TG-Ab (*r* = −0.15, *p* = 0.25), TSH (*r* = −0.014, *p* = 0.09) and FT4 (*r* = 0.13, *p* = 0.32).

## 4. Discussion

The main finding of this study was that elderly patients with vitamin D deficiency had a significantly higher prevalence of AT compared to elderly patients with vitamin D sufficiency. These results were in agreement with previous studies in younger populations [8]. Further, 25(OH) vitamin D levels were significantly correlated with FT3 and TPO-Ab levels in the cohort of patients with AT.

The prevalence of vitamin D deficiency (70%) was high in our elderly population, although this value is consistent with the results reported in European studies assessing vitamin D deficiency in the elderly [15,16,17,18]. This strict interaction between vitamin D deficiency and AT has been also suggested by Tamer et al. [7], who demonstrated an increased prevalence of vitamin D deficiency (25(OH) vitamin D ≤ 10 ng/mL) in patients with AT. These findings can be explained by molecular evidence indicating a role of vitamin D in the pathogenesis of autoimmune diseases. Vitamin D deficiency has been recently considered as an environmental trigger of AT [7,9,19].

The Vitamin D receptor (VDR) is an intracellular receptor that is expressed by human immune cells, such as macrophages, dendritic cells, and T and B lymphocytes, although the main target is represented by dendritic cells (DC) [20,21]. The immunomodulatory action of 1,25(OH)_2_D is expressed thanks to the binding of 1,25(OH)_2_D to its receptor [22]. In particular 1,25(OH)_2_D inhibits DC maturation and the production of DC-derived cytokines, such as IL-12 and IL-23. This effect of 1,25(OH)_2_D prompts the T cells to differentiate towards the Th2 phenotype, rather than to the Th1 and Th17 phenotypes [23]. Moreover, vitamin D promotes the release of DC-derived IL-10 release which has tolerogenic properties and reduces the production of inflammatory Th1 cytokines, such as IL-2 and IFN-γ, which promote cell-mediated cytotoxicity responsible for thyroid destruction in AT [23]. Finally, several functional polymorphisms in the VDR gene or vitamin D binding protein (VDB) have been involved in the pathogenesis of AT. In particular, the polymorphism of VDB has been associated with Graves’ disease both in the Polish and Japanese populations [24,25]. A meta-analysis of eight studies suggested that the Bsml or Taql VDR polymorphism may be involved in the pathogenesis of AT [26]. Polymorphisms of the CYP27B1 gene have been also reported to have a role in the pathogenesis of AT [27]. 

The degree of vitamin D deficiency has been reported to have a tight relationship with antibody levels and with thyroid hormones, thus suggesting that vitamin D levels may have a direct role in determining the grade of severity of autoimmune disease and of the consequent hypothyroidism [28,29]. In our study we found that 25(OH) vitamin D levels significantly correlated with TPO-Ab and FT3, while we failed to find any association of 25(OH) vitamin D levels with TG-Ab, FT4 and TSH. 

## 5. Conclusions

In conclusion, within the limitations of a cross-sectional design, the present study shows that vitamin D deficiency was associated with AT in the elderly. Future longitudinal cohort studies along with prospective interventional trials may contribute to better clarifying if vitamin D supplementation may have a role in the prevention and/or therapy of AT in the elderly.

## Figures and Tables

**Table 1:** Clinical and hormonal data stratified according to the vitamin D levels (mean ± SD).

Parameters	Total (*n* = 168)	Vitamin D Deficiency (25(OH) Vitamin D < 20 ng/mL) (*n* = 116)	Vitamin D Sufficiency (25(OH) Vitamin D ≥ 20 ng/mL) (*n* = 52)	*p*-Value
Age (years)	81.6 ± 9.4	82.8 ± 8.4	78.8 ± 10.8	0.005
25(OH) vitamin D (ng/mL)	16.4 ± 15.6	7.6 ± 4.0	33.3 ± 19.6	<0.01
BMI (kg/m^2^)	29.8 ± 15.2	28.7 ± 8.6	32.4 ± 24.1	0.12
Glycemia (mg/dL)	102.4 ± 32.2	104.2 ± 33.2	101.5 ± 31.8	0.31
Creatinine (mg/dL)	1.1 ± 0.6	1.1 ± 0.7	0.9 ± 0.3	0.04
AST (UI/L)	19.9 ± 7.9	19.1 ± 6.4	21.7 ± 10.4	0.30
ALT (UI/L)	17.6 ± 11.7	16.9 ± 10.7	19.1 ± 13.7	0.13
Total Cholesterol (mg/dL)	173.8 ± 44.6	167.0 ± 41.9	176.9 ± 45.6	0.09
Triglycerides (mg/dL)	113.9 ± 7.8	104.7 ± 40.1	118.1 ± 82.1	0.13
TSH (mcUI/mL)	2.1 ± 0.9	2.1 ± 1.9	1.8 ± 1.3	0.14
FT3 (pg/mL)	3.3 ± 0.4	3.7 ± 1.0	4.1 ± 1.6	0.16
FT4 (ng/dL)	15.1 ± 4.7	15.3 ± 5.3	14.7 ± 3.1	0.33
TPO-Ab (IU/mL)	105 ± 45.9	117 ± 57.6	69.1 ± 19.8	0.01
TG-Ab (IU/mL)	121 ± 47.0	128 ± 55.7	101 ± 27.4	0.08
AT prevalence (%)	23	28	8	0.02

## References

[1] DeLuca H.F. (2004). Overview of general physiologic features and functions of vitamin D. Am. J. Clin. Nutr..

[2] Holick M.F. (2007). Vitamin D deficiency. N. Engl. J. Med..

[3] Christakos S., Dhawan P., Verstuyf A., Verlinden L., Carmeliet G. (2016). Vitamin D: Metabolism, Molecular Mechanism of Action, and Pleiotropic Effects. Physiol. Rev..

[4] Basit S. (2013). Vitamin D in health and disease: A literature review. Br. J. Biomed. Sci..

[5] Muscogiuri G., Mitri J., Mathieu C., Badenhoop K., Tamer G., Orio F., Mezza T., Vieth R., Colao A., Pittas A. (2014). Mechanisms in endocrinology: Vitamin D as a potential contributor in endocrine health and disease. Eur. J. Endocrinol..

[6] McDonnell D.P., Pike J.W., O’Malley B.W. (1988). The vitamin D receptor: A primitive steroid receptor related to thyroid hormone receptor. J. Steroid Biochem..

[7] Tamer G., Arik S., Tamer I., Coksert D. (2011). Relative vitamin D insufficiency in Hashimoto’s thyroiditis. Thyroid.

[8] Mazokopakis E.E., Kotsiris D.A. (2014). Hashimoto’s autoimmune thyroiditis and vitamin D deficiency. Current aspects. Hell. J. Nucl. Med..

[9] Lacka K., Maciejewski A. (2013). Vitamin D in the etiopathogenesis of autoimmune thyroiditis. Pol. Merkur. Lekarski.

[10] Goswami R., Marwaha R.K., Gupta N., Tandon N., Sreenivas V., Tomar N., Ray D., Kanwar R., Agarwal R. (2009). Prevalence of vitamin D deficiency and its relationship with thyroid autoimmunity in Asian Indians: A community-based survey. Br. J. Nutr..

[11] Effraimidis G., Badenhoop K., Tijssen J.G.P., Wiersinga W.M. (2012). Vitamin D deficiency is not associated with early stages of thyroid autoimmunity. Eur. J. Endocrinol..

[12] Muscogiuri G., Palomba S., Caggiano M., Tafuri D., Colao A., Orio F. (2015). Low 25(OH) vitamin D levels are associated with autoimmune thyroid disease in polycystic ovary syndrome. Endocrine.

[13] Heijboer A.C., Blankenstein M.A., Kema I.P., Buijs M.M. (2012). Accuracy of 6 routine 25-hydroxyvitamin D assays: Influence of vitamin D binding protein concentration. Clin. Chem..

[14] Holick M.F., Binkley N.C., Bischoff-Ferrari H.A., Gordon C.M., Hanley D.A., Heaney R.P., Murad M.H., Weaver C.M., Endocrine Society (2011). Evaluation, treatment, and prevention of vitamin D deficiency: An Endocrine Society clinical practice guideline. J. Clin. Endocrinol. Metab..

[15] Mosekilde L. (2005). Vitamin D and the elderly. Clin. Endocrinol. (Oxf.).

[16] Omdahl J.L., Garry P.J., Hunsaker L.A., Hunt W.C., Goodwin J.S. (1982). Nutritional status in a healthy elderly population: Vitamin D. Am. J. Clin. Nutr..

[17] McKenna M.J. (1992). Differences in vitamin D status between countries in young adults and the elderly. Am. J. Med..

[18] Holick M.F. (1995). Environmental factors that influence the cutaneous production of vitamin D. Am. J. Clin. Nutr..

[19] Muscogiuri G., Tirabassi G., Bizzaro G., Orio F., Paschou S.A., Vryonidou A., Balercia G., Shoenfeld Y., Colao A. (2015). Vitamin D and thyroid disease: To D or not to D?. Eur. J. Clin. Nutr..

[20] Baeke F., Etten E.V., Overbergh L., Mathieu C. (2007). Vitamin D_3_ and the immune system: Maintaining the balance in health and disease. Nutr. Res. Rev..

[21] Karthaus N., van Spriel A.B., Looman M.W., Chen S., Spilgies L.M., Lieben L., Carmeliet G., Ansems M., Adema G.J. (2014). Vitamin D controls murine and human plasmacytoid dendritic cell function. J. Investig. Dermatol..

[22] Mathieu C. (2011). Vitamin D and the immune system: Getting it right. IBMS BoneKEy.

[23] Baeke F., Takiishi T., Korf H., Gysemans C., Mathieu C. (2010). Vitamin D: Modulator of the immune system. Curr. Opin. Pharmacol..

[24] Kurylowicz A., Ramos-Lopez E., Bednarczuk T., Badenhoop K. (2006). Vitamin D-binding protein (DBP) gene polymorphism is associated with Graves’ disease and the vitamin D status in a Polish population study. Exp. Clin. Endocrinol. Diabetes.

[25] Ban Y., Ban Y., Taniyama M., Katagiri T. (2000). Vitamin D receptor initiation codon polymorphism in Japanese patients with Graves’ disease. Thyroid.

[26] Feng M., Li H., Chen S.F., Li W.F., Zhang F.B. (2013). Polymorphisms in the vitamin D receptor gene and risk of autoimmune thyroid disease: A meta-analysis. Endocrine.

[27] Pani M.A., Regulla K., Segni M., Krause M., Hofmann S., Hufner M., Herwig J., Pasquino A.M., Usadel K.H., Badenhoop K. (2002). Vitamin D 1α-hydroxylase (CYP1α) polymorphism in Graves’ disease, Hashimoto’s Thyroiditis and type 1 diabetes mellitus. Eur. J. Endocrinol..

[28] Bozkurt N.C., Karbek B., Ucan B., Sahin M., Cakal E., Ozbek M., Delibasi T. (2013). The association between severity of vitamin D deficiency and Hashimoto’s thyroiditis. Endocr. Pract..

[29] Mackawy A.M., Al-Ayed B.M., Al-Rashidi B.M. (2013). Vitamin d deficiency and its association with thyroid disease. Int. J. Health Sci. (Qassim).

